# Regeneration of Aluminum Matrix Composite Reinforced by SiC_p_ and GC_sf_ Using Gas Tungsten Arc Welding Technology

**DOI:** 10.3390/ma14216410

**Published:** 2021-10-26

**Authors:** Katarzyna Łyczkowska, Janusz Adamiec, Anna Janina Dolata, Maciej Dyzia, Jakub Wieczorek

**Affiliations:** Faculty of Materials Engineering, Silesian University of Technology, ul. Krasińskiego 8, 40-019 Katowice, Poland; janusz.adamiec@polsl.pl (J.A.); anna.dolata@polsl.pl (A.J.D.); maciej.dyzia@polsl.pl (M.D.); jakub.wieczorek@polsl.pl (J.W.)

**Keywords:** aluminum matrix composites, silicon carbide, glassy carbon, GTAW welding, tribological test

## Abstract

The main motivation behind the presented research was the regeneration of the damaged surface of composite materials. The testing of melting and pad welding of the composite surface by Gas Tungsten Arc Welding (GTAW) with alternating current (AC) were carried out. The material of investigation was an AlSi12/SiCp + GCsf hybrid composite made by a centrifugal casting process. The composite was reinforced with 5 wt.% of silicon carbide particles and 5 wt.% of glassy carbon spheres. The composites were investigated in tribological tests. It was found that there was a possibility for modification or regeneration of the surface with pad welding technology. Recommended for the repairs was the pad welding method with filler metal with a chemical composition similar to the aluminum matrix composite (ISO 18273 S Al4047A (AlSi12 [A])). The surface of the pad welding was characterized by the correct structure with visible SiCp. No gases or pores were observed in the pad welding; this was due to a better homogeneity of the silicon carbide (SiCp) distribution in the composite and better filling spaces between liquid metal particles in comparison to the base material. Based on the tribological tests, it was found that the lowest wear was observed for the composite surface after pad welding. This was related to the small number of reinforcing particles and their agreeable bonding with the matrix. The plastic deformation of the Al matrix and scratching by worn particles were a dominant wear mechanism of the surface.

## 1. Introduction

Aluminum matrix composites (AMCs) are common materials that are applied in many fields of industry, such as automotive, aerospace, electricity, chemical, etc., due to their unique properties [1,2,3]. The most used reinforcement phases are oxides (Al_2_O_3_, ZrO_2_, SiO_2_), carbides (SiC, TiC, B_4_C, ZrC), and nitrides (Si_3_N_4_) [4]. The presence of these phases in strict proportion (normally between 5 and 30 wt.%) leads to an increase in material properties [1,4,5,6,7].

Aluminum matrix composites reinforced by silicon carbide (SiC) are a material solution successfully applied in the automotive field [8,9,10]. The popularity of these composites is due to a number of their properties, such as wear resistance, stiffness, compressive strength, low density, low coefficient of thermal expansion, good casting properties, and low production costs [11,12]. This kind of composite is widely used in tribological conditions where material is constantly subjected to variable loads and high-temperature conditions. For this reason, AMCs reinforced with SiC particles need to show high stability of tribological properties regardless of changing working conditions. These special types of materials are composites used in conditions where the surface is constantly subjected to wear, such as cylinder liners, brake disk, or pistons of engines or compressors [13,14,15,16,17].

Generally, in the case of wear, the whole element has to be replaced even if only a small part of the material surface is damaged by external conditions (e.g., pitting or scuffing mechanisms). The replacement of the whole element causes negative economic and environmental impacts because there remains a lack of effective methods of recycling aluminum matrix composites reinforced by silicon carbide [18,19,20,21].

There are various reports on the methods of welding aluminum composite castings. Based on the literature review, it can be stated that the most popular and promising method of Al/SiCp joining is Friction Stir Welding (FSW). Kurtyka et al. [22,23,24] described that one of the results of FSW process implementation can be a significant improvement in the distribution of the reinforcing phase particles. This process influences the mechanical properties of the composite and, compared to the starting material, it allows for an increase of approximately 40% of compressive strength and 30% of hardness. All the results confirm the effectiveness of the FSW method for joining aluminum matrix composites reinforced by SiC particles or other types of ceramic phases. This method is also intended for the joining of AMCs with different types of ceramic reinforcement [25].

Due to its technological solution, the FSW method is recommended for welding elements with specific dimensions, which significantly limits the regeneration possibilities of damaged areas.

The next part of the literature analysis focuses on bonding and remelting through other advanced welding technologies—Electron Beam Welding (EBW) and Laser Beam Welding (LBW). Wang at al. [26] described welding of 101Al/SiC_p_ composites by EBW technology. According to these studies, a small quantity of brittle Al_4_C_3_ compound and a single Si phase were generated in the welded joint. However, the authors proved that the interfacial reaction between SiC particles and the Al matrix could be greatly suppressed by high welding speed and low heat input. Based on the research, it was confirmed that LBW welding and hardfacing enables the production of welding joints with the required properties.

Studies conducted by Dahotre et al. [27] showed that the alloy matrix composites reinforced with 10 and 20 vol.% of SiC particulates were more readily welded by LBW. The opposite effect was observed in the composite materials where the fusion zone contained the fully melted matrix and the fully reacted SiC reinforcement, and where the heat-affected zone contained the partially melted matrix and the nearly unreacted SiC particles. Moreover, the authors showed that increasing the SiC content from 0 to 20% caused a decrease in the reflection of the laser beam and an increase in melt viscosity. This was potentially caused by an increasing amount of Al_4_C_3_ compound [28].

In turn, Wang et al. [29] described the results of an investigation with the use of micro-nano (Al–Si–Cu)–Ti foils as filler metal. The high-performance joints of aluminum matrix composites with high SiC particle content (Al-MMC_s_/60% SiC_p_) were observed. Moreover, the beneficial effect of adding Ti into the filler metal on improving wettability between SiC particles and the metallic brazed seam was confirmed.

Equally satisfactory results of Al/SiC_p_ joining were obtained using the plasma spray process [30], welding by oxy-acetylene [31], and soldering and gluing [32].

Based on the literature review, it was found that more and more technologies are helping to obtain a permanent joint of Al/SiC_p_ composites. However, it should be mentioned that the main problem during welding is the appearance of the Al_4_C_3_ phase, which may lead to a reduction in the strength properties of materials [22,27,29]. It is important to use strictly defined parameters of welding and pad welding that will limit the formation of the unfavorable Al_4_C_3_ phase.

Unfortunately, many technologies are too expensive and complicated to use, hence the need to develop a technology for the surfacing and regeneration of the damaged composite surface that will be relatively easy and available. One of the promising and still-developing technologies used for the regeneration of different kinds of materials is the Gas Tungsten Arc Welding (GTAW) method that helps to join metal–ceramic composites by GTAW DC welding, although SiC reinforcing particles have a much lower thermal conductivity than a metal matrix. It has also been found that the conditions favor the precipitation processes in the solidified mixture of the fused composite matrix and the additive material, which allows them to be effectively joined. These obtained results [33,34] are the main motivation for future work on the development of the GTAW method, which will allow for the regeneration of the composite at low cost.

The available research shows that there are some articles on Al/SiC_p_ composites, however there is no information on the surface regeneration of the Al/SiC_p_ composites with the addition of glassy carbon. It is necessary to conduct research that will contribute to the development an effective technology for the regeneration of the damaged piston surface of aluminum matrix composites reinforced by SiC and glassy carbon.

## 2. Materials and Methods

The material of investigation was a composite based on EN AC-48000 alloy (AlSi12CuNiMg) made by a centrifugal casting process. The material was reinforced with 5 wt.% of silicon carbide particles (SiC_p_) of average size in the range of 30–70 µm, and 5 wt.% of spherical glassy carbon (GC_sf_) of average size in the range of 5–15 µm.

**Figure 1:**
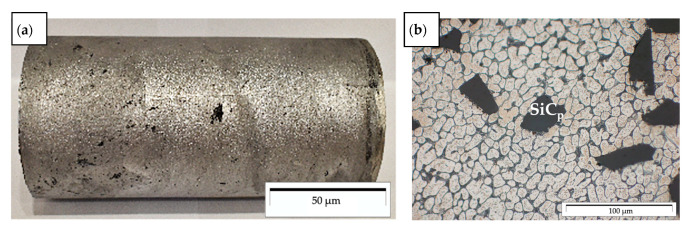
AlSi12/SiC_p_ + GC_sf_ composite sleeves: (**a**) view of a representative cast of the composite sleeve; (**b**) microstructure of the SiC_p_ particle-rich region.

**Figure 2:**
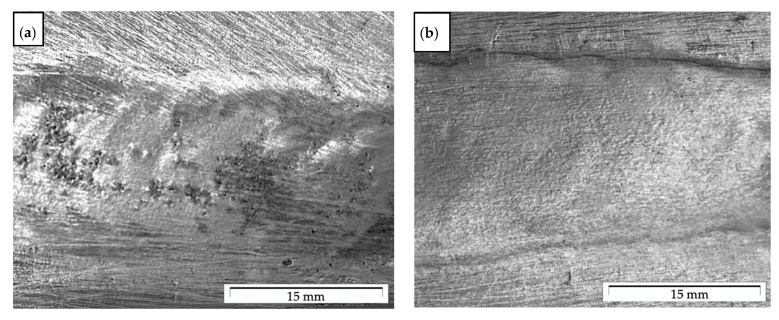
Face of the AlSi12/SiC_p_ + GC_sf_ composite weld: (**a**) remelting; (**b**) pad welding.

In the first stage, composite suspensions were prepared by stir casting in an autoclave furnace with a moving graphite stirrer system, according to the procedure described in [7,14]. In the second stage, heated composite suspension was cast into the rotating mold (d = 60 mm; ω = 3000 rpm), according to the method described in previous own works [12,13]. An example of a cast of composite sleeves and its microstructure in the outer area is shown in Figure 1. Due to the gradient structure in this type of casting [12], remelting and pad welding tests were carried out on the outer surface of the composite sleeve. The welding test was carried out by the GTAW method using filler material AlSi12 with a diameter of 2 mm, and argon as the inert shielding gas, at a flow rate of 10 l/min. The parameters of welding technology are shown in Table 1. The remelting process was carried out with 120 A alternating current, and the pad welding with 140 A alternating current. The results of remelting and pad welding are shown in Figure 2. The material was not preheated prior to the pad welding process.

**Figure 3:**
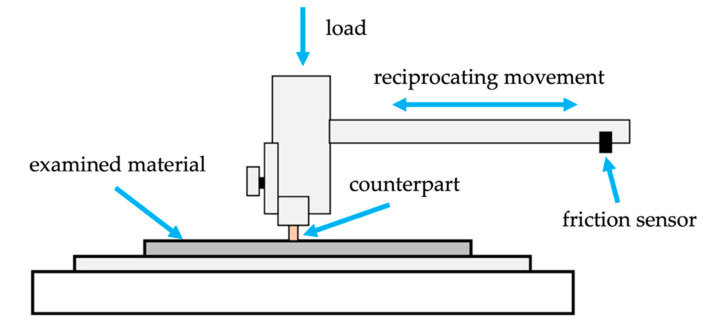
Scheme of a device for tribological testing in the reciprocating friction system.

The tribological investigations were performed by reciprocating movement in ambient condition and without lubrication. A speed of 4 m/min, a load of 1.5 kg, and a distance of 500 m were applied as the main tribological parameters. The GJL300 iron was used as a typical material for a tribological partner. The counterpart was in the form of a pin with 6 mm diameter. The surfaces before testing were grinded by using sandpaper with 1000 gradation. The scheme of the device is shown in Figure 3.

The wear surfaces were analysed on a MicroProf 3000, FRT optical profilometer, FRT GmbH, Bergisch Gladbach (Germany). Based on these results, roughness values (Ra), root mean square (Rq), and average maximum height of the surface (Rz) of wear were achieved.

The metallographic examinations were conducted using an Olympus GX71, Warsaw (Poland) light microscope (LM) at magnifications of up to 500×. The structure of the surface after the welding and tribological tests were examined under the scanning electron microscopes (SEM) JEOL JCM-6000 Neoscope II, Tokyo (Japan), and Hitachi S-4200, Krefeld (Germany). Images were recorded in the secondary electron mode at a magnification of 1000× and at a voltage accelerating the electron beam to 15 keV.

## 3. Results and Discussion

Aluminum matrix composites made by centrifugal casting are characterized by a complex and heterogeneous structure. The differences depend on the casting structure of the aluminum alloy and the inhomogeneous distribution of reinforcing particles as a result of the centrifugal force during the casting process (Figure 1b).

**Figure 4:**
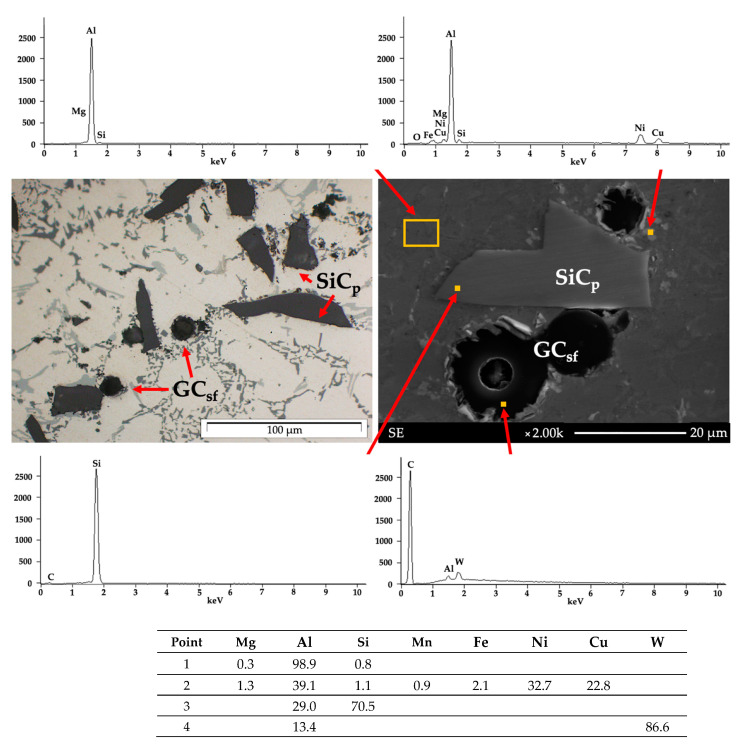
The microstructure and results of EDS analysis of AlSi12/SiC_p_ + GC_sf_ composite.

The study materials are characterized by AlSi12/SiC_p_ + GC_sf_, made of silicon eutectic (α + Si), solid solution α, primary silicon crystals, and intermetallic crystal phases. Spherical glassy carbon particles were distributed in the matrix, mainly in the middle zone, and SiC particles were distributed in the surface zone (Figure 4). This particle distribution is characteristic of centrifugal casting, and it is a result of the density of the individual phases.

**Figure 5:**
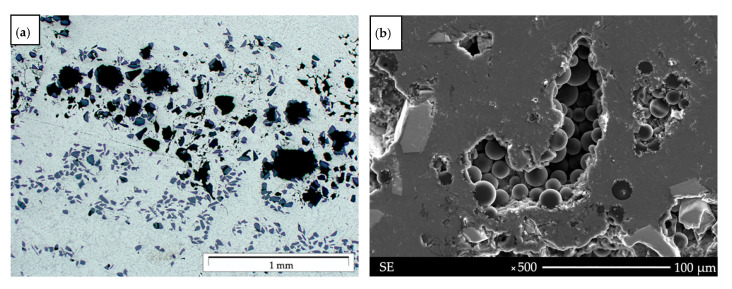
The microstructure of AlSi12/SiC_p_ + GC_sf_ composites: (**a**) gas porosity in SiC_p_ area, (**b**) pores and voids with GC_sf_ in areas of inhomogeneous distribution of particles.

A significant problem was the existence of many pores and gas bubbles, especially in the concentration of the reinforcement phase, which is related to high surface tension and the inability to fully penetrate space mainly in the liquid phase during crystallization (Figure 5). The second phenomenon determining the formation of voids is excessively found in fluid of low pressure and caused by the lack of supply of liquid metal to fill the space between the reinforcing particles. This phenomenon was described, among others, in works [35,36]. Based on the results, remelting and pad welding are one of the solutions to reduce the porosity of the subsurface zone, which works in the wear systems described.

The results of the visual examination of remelting revealed a uniform weld face with some small discontinuities. This is related to the presence of pores and the inhomogeneous distribution of the reinforcement phases, mainly for SiC_p_ (Figure 2a). A similar weld face was observed in the surface of the pad welding (Figure 2b). The weld face was smooth and uniform, which indicated the correct selection of the pad welding parameters. No pores and bladders were observed on the surface. On this basis, it was concluded that the welding filler wire for pad welding (AlSi12) increased the area of the liquid metal pool and filled the space between the reinforcing particles and the aluminum matrix better. It is also important to reduce the number of reinforcing particle units of volume of the liquid metal pool.

The examination of the chemical composition of the AlSi12/SiC_p_ + GC_sf_ composites (pt. 1) by the EDS method in individual areas revealed SiC (pt. 3) and spherical glassy carbon (pt. 4). Fe-containing phases and Ni- and Cu-containing phases (pt. 2) were also disclosed. 

**Figure 6:**
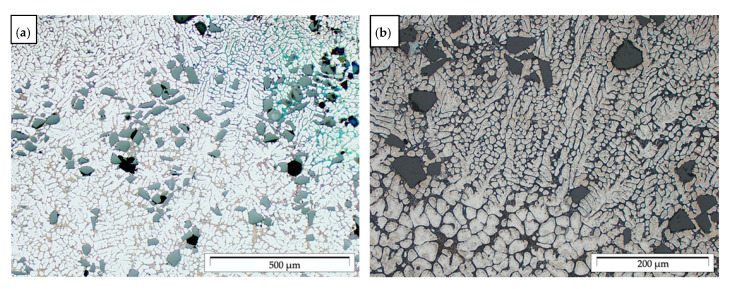
The microstructure of tested material after modification using welding techniques: (**a**) remelting structure with a visible reinforcement phase (SiC_p_) and small number of pores; (**b**) pad welding with visible fusion zone and SiC_p_.

The microstructure of the cross section with an orientation perpendicular to the remelting and pad welding direction indicated that pad welding had a positive impact on the quality of the subsurface layer. The reason for this is the homogenization of the SiC_p_ reinforcement phases and the reduction in the number of pores in the composite (Figure 6a). In this area, the GC_sf_ was not observed, providing information about the segregation of GC_sf_ particles in the inner zone of the sleeve. Single reinforcing particles were revealed in the pad welding, however no pores and voids were found (Figure 6b). This indicates a properly selected pad welding technology.

**Figure 7:**
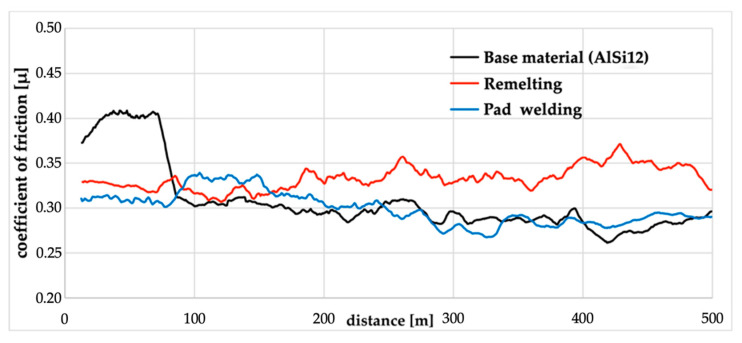
The variation of friction coefficient tested for base material (AlSi12/SiC_p_ + GC_sf_) and composites repaired by remelting and pad welding processes.

In order to determine the operating conditions of the repaired composite elements, (e.g., low-loaded pistons engines [34]), the resistance tests of tribological wear in a reciprocating system were carried out. This is a typical friction system found in compressors and reciprocating internal combustion engines. The analysis of the coefficient of friction for materials without any modification indicated that the coefficient was constant over the entire range of the experiment (over a distance of 500 m) and was on average equal to 0.33 (Figure 7). The analysis of the remelting surface showed that in the initial period (approximately 150 m), the friction coefficient increased significantly to a value of up to 0.4.

**Figure 8:**
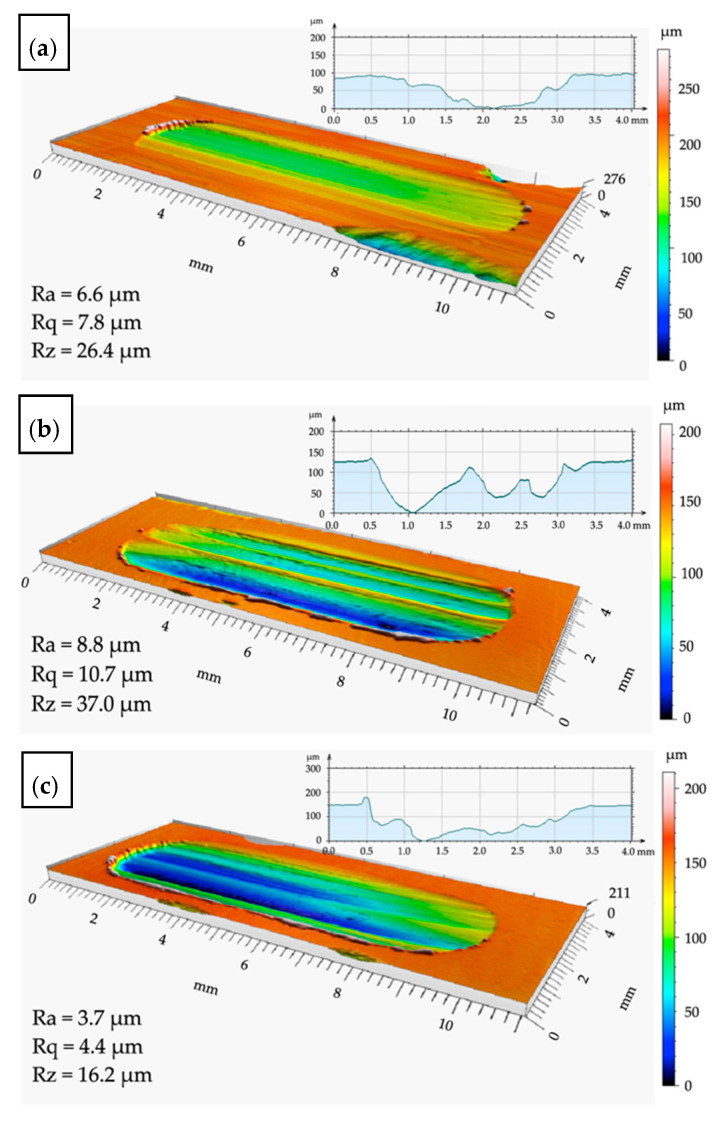
View of the wear surface after tribological tests: (**a**) surface of the base material (AlSi12/SiC_p_ + GC_sf_); (**b**) remelting area; (**c**) pad welding area.

For the remelted material, slightly higher values of surface roughness parameters were obtained, Ra = 8.8 µm, Rq = 10.7 µm, and Rz = 37.0 µm, and the wear depth was approximately 130 µm (Figure 8b). This is a surface wear mechanism similar to that previously described, however, the cross-sectional profile indicates the scratching and ridging mechanisms. The profile of the worn surface depends on the behavior of individual SiC particles (Figure 8b). In the remelting process, the friction effect is reduced. It should be stated that the remelting of the composite surface does not yield a positive result for research.

**Figure 9:**
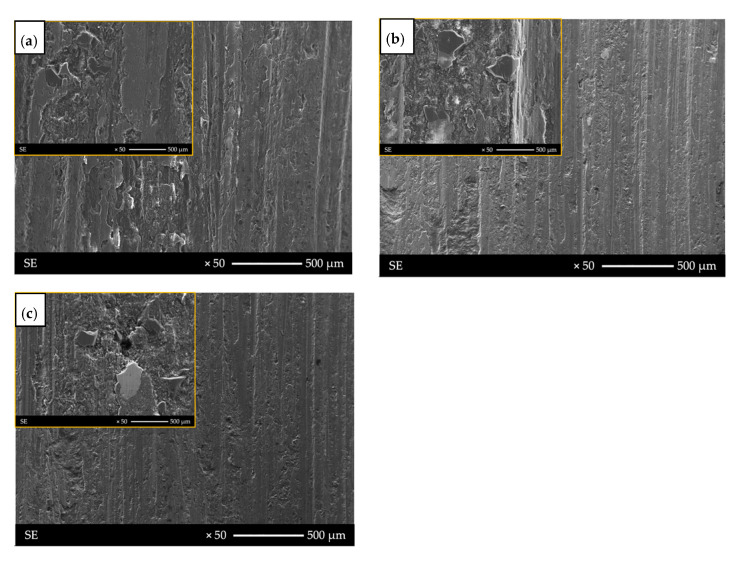
Surfaces after tribological test: (**a**) base material (AlSi12/SiCp + GCsf); (**b**) remelting area with visible SiCp; (**c**) pad welding area with single reinforcing particles.

**Figure 10:**
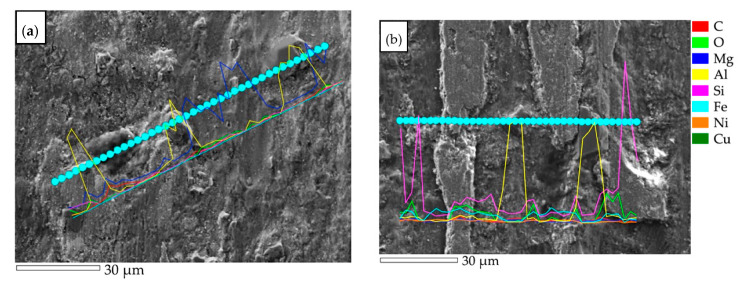

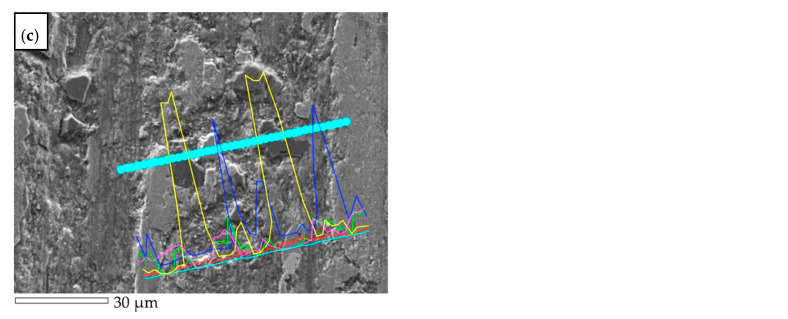
SEM–EDS line analysis after tribological test: (**a**) base material (AlSi12/SiC_p_ + GC_sf_); (**b**) remelting area; (**c**) pad welding area.

This was related to the lapping process of the surface as a result of pulling out single SiC_p_ particles (Figure 9b). After this stage, the coefficient of friction value stabilized at 0.3, which is lower than for the base material. Similar values were obtained for the surface of the pad welding, which indicates that after the abrasion step, the main friction surface becomes the matrix. The coefficient of friction value for pad welding was 0.3. Single SiC_p_ particles were observed on the wear surface of the pad welding (Figure 9c and Figure 10).

**Figure 11:**
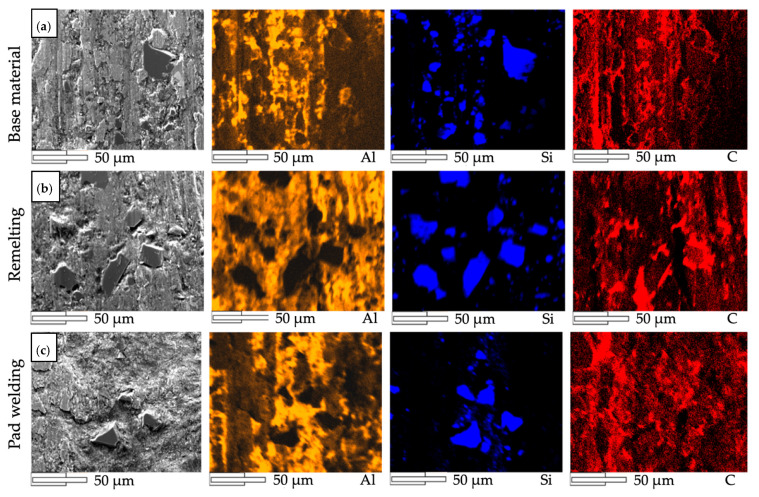
X-ray spectroscopy (EDS) mapping analysis of the wear track: (**a**) base material; (**b**) remelting area; (**c**) pad welding area.

The profilometric observations of the wear process were supplemented by metallographic analysis (Figure 9 and Figure 10). In the case of the base material, the surface development indices Ra, Rq, and Rz were the highest, with Ra = 6.6 µm, Rq = 7.8 µm, and Rz = 26.4 µm, respectively. The profile valley depth of the wear was approximately 150 µm (Figure 8a). These results were confirmed by the wear mechanism observed in metallographic tests (Figure 9 and Figure 10). The wear of the surface was determined by reinforcing SiC particles, which furrowed after being pulled out of the matrix. In the next stage, the SiC particles were pushed into the matrix and the furrow was obliterated as a result of the plastic deformation of the Al matrix (Figure 9a, Figure 10a and Figure 11a).

The best results were obtained for the composite surface after pad welding (Figure 10c and Figure 11c). This was also confirmed by the surface parameters index, which were Ra = 3.7 µm, Rq = 4.4 µm, and Rz = 16.2 µm, and the valley depth of the wear was approximately 100 µm (Figure 8c). It was found that, due to the small number of reinforcing particles (Figure 6 and Figure 11c) and their agreeable bonding with the matrix (Figure 9c), no ridging or particle pull-out phenomena occur. There was even wear as a result of the plastic deformation of the matrix (Figure 11c) and surface scratching (Figure 9c).

On this basis, it can be assumed that a good technology for layer modification and for the repair or regeneration of elements working in reciprocating friction wear conditions is pad welding by GTAW method, with filler metal with a chemical composition suitable to the matrix.

## 4. Conclusions

Based on the research and the analysis of the obtained results, the following conclusions were drawn:

1. It is possible to modify or repair the surface of AlSi12/SiC_p_ + GC_sf_ aluminum matrix composites reinforced by SiC_p_/GC_sf_ made by centrifugal casting. The application of GTAW method with filler metal characterized by a chemical composition similar to the aluminum metal matrix composite is a confirmed method to achieve pad welding with the required properties. The process should be carried out in argon gas, at a flow rate of 10 l/min, and with an alternating current from 120 to 140 A.
2. The surface of the composite sleeve after remelting is characterized by the correct structure, in which the SiC_p_/GC_sf_ reinforcing particles are observed. A much lower porosity of the remelted zone was found. This is due to a better homogeneity of the SiC_p_ distribution in the composite and better filling spaces between liquid metal particles in comparison to the base material.
3. Single SiC_p_ particles were observed in the area of pad welding made with AlSi12 filler metal, this results from the major volume of the matrix in the liquid metal pool. No pores or gases were observed in the pad welding, which confirms the correct repair process.
4. The surface of the composite after the pad welding process is characterized by similar tribological properties as the base material, while the pad welding under the same conditions shows a lower degree of wear. This is due to a smaller number of reinforcing particles that cause the surface to be furrowed and the plastic to deformation.

## Figures and Tables

**Table 1:** The parameters of remelting and pad welding of AlSi12/SiC_p_ + GC_sf_ composite.

Composite Material	Process	Welding Current (A)	Voltage (V)	Welding Speed (cm/min)	Gas Flow Rate (L/min)
AlSi12/SiC_p_ + GC_sf_	Remelting	120	14	20	10
	Pad welding	140	16	20	10

## Data Availability

The data supporting reported results are not stored in any publicly archived datasets. The readers can contact the corresponding author for any further clarification of the obtained results.
